# A Fire Department Community Health Intervention to Prevent Carbon Monoxide Poisoning Following a Hurricane

**DOI:** 10.1371/currents.dis.b7b37e23941192c412959915652792e4

**Published:** 2014-02-18

**Authors:** Matthew Levy, J Lee Jenkins, Kevin Seaman

**Affiliations:** Department of Emergency Medicine, Johns Hopkins University School of Medicine, Baltimore, Maryland, USA

## Abstract

Portable generators are commonly used during electrical service interruptions that occur following large storms such as hurricanes. Nearly all portable generators use carbon based fuels and produce deadly carbon monoxide gas. Despite universal warnings to operate these generators outside only, the improper placement of generators makes these devices the leading cause of engine related carbon monoxide deaths in the United States. The medical literature reports many cases of Carbon Monoxide (CO) toxicity associated with generator use following hurricanes and other weather events. This paper describes how Howard County, Maryland Fire and Rescue (HCFR) Services implemented a public education program that focused on prevention of Carbon Monoxide poisoning from portable generator use in the wake of events where electrical service interruptions occurred or had the potential to occur. A major challenge faced was communication with those members of the population who were almost completely dependent upon electronic and wireless technologies and were without redundancies. HCFR utilized several tactics to overcome this challenge including helicopter based surveillance and the use of geocoded information from the electrical service provider to identify outage areas. Once outage areas were identified, HCFR personnel conducted a door-to-door canvasing of effected communities, assessing for hazards and distributing information flyers about the dangers of generator use. This effort represents one of the first reported examples of a community-based endeavor by a fire department to provide proactive interventions designed to prevent carbon monoxide illness.

## Introduction

Natural disasters, including severe storms, often cause interruptions in a region’s critical infrastructure. The loss of electrical power in particular results in delays in the recovery process. These delays are known to contribute to the cause of a variety of illnesses and injuries.^1^^,^ 2 Portable power generators are frequently utilized to provide electricity to essential items in residential homes. These generators are available in a variety of sizes, are relatively inexpensive to purchase and easily found at home improvement stores. Homeowners constitute the largest group of end users of portable power generators.^3^

A paradox exists between the ubiquitous availability of portable generators and their associated dangers. 98% of portable generators on the consumer market are fueled by carbon-based fuels.^3^ The United States Consumer Products Safety Commission (CPSC) states that majority of carbon monoxide related deaths from engine driven tools in the U.S. are related to the improper use of portable generators.^4^ In addition, the medical literature contains multiple accounts of Carbon Monoxide (CO) toxicity in the immediate post-hurricane period as a result of gas-powered generators. 5 ^,^ 6 ^,^ 7 ^,^ 8

CO is the leading cause of accidental poisoning deaths in the US and accounts for over 500 fatalities, 20,000 emergency department visits and 4,000 hospital admissions each year.^9^ During a 5 year period from 1999-2004, Maryland had a total of 46 deaths due to accidental CO exposure, for a rate of 1.43 deaths per million persons per year, compared with a national rate of 1.53 deaths per million per year.^10^ This data further reaffirm carbon monoxide’s notorious reputation of being a killer.

Recognition of CO is often challenging given its colorless and odorless characteristics.^10^ In addition, the symptoms of CO poisoning mimic so many other illnesses that CO poisoning is often difficult to diagnose. This report describes a county fire department's experience with CO exposures in the aftermath of two hurricanes, as well as the fire department-based public health intervention and prevention program implemented to mitigate future exposures.

## Recent CO Cases in Howard County Highlighting the Problem

August, 2011 following Hurricane Irene

Three patients were discovered unresponsive in a detached single family dwelling. Fire Department personnel assessed the CO levels in the home to be toxic with readings greater than 1500ppm by the by the ITX Multi-Gas Monitor (Industrial Scientific Corporation, Oakdale, Pennsylvania USA). The National Institute of Occupational Safety and Health states that Immediately Dangerous to Life or Health (IDLH) level for inhaled Carbon Monoxide is 1200 ppm.^9^ Point of care co-oximetry levels using a RAD 57 Signal Extraction Pulse CO-Oximeter (Massimo Corporation, Irvine, California, USA) were 30% SpCO for Patient 1 and 35% SpCO for Patient 2. Both patients were transported to the regional Hyperbaric Specialty Center for treatment. Tragically, Patient 3 was deemed a fatality as a direct result of the CO exposure. Investigators determined that a gasoline generator running inside the home near the garage was the cause of the deadly CO levels.

September, 2011 following Hurricane Irene

Three patients called 911 on the evening after returning from a job site shortly after Hurricane Irene. They had been working earlier in the day for approximately four hours in a basement where a gasoline-powered pump was running. All three patients were experiencing headache and dizziness upon FD arrival. RAD 57 (Masimo Corporation, Irvine, California, USA) measurements >20% SpCO were detected in all three patients several hours after reported exposure. These patients were transported to the hospital. A crew was sent to the exposure site and discovered residual CO levels of 50 ppm (ITX Multi-Gas Monitor Industrial Scientific Corporation, Oakdale, Pennsylvania USA) several hours after the pump had been turned off.

October, 2012 following Hurricane Sandy

In the early morning hours following Hurricane Sandy, HCFR crews responded to a single family detached residence for of a report of multiple patients with symptoms of CO poisoning. A generator had been running for approximately one day and ambient CO levels in the house were measured at over 1500 ppm (ITX Multi-Gas Monitor (Industrial Scientific Corporation, Oakdale, Pennsylvania USA). Patient 1 was unconscious and evacuated from the residence and started on high flow oxygen. Patient 1's RAD 57 (Masimo Corporation, Irvine, California, USA) measurement 20 minutes after removal from the house was 35% SpCO. Patient 2 presented awake and alert with symptoms including headache, chest tightness and nausea. Patient 2 had an initial RAD 57 measurement was 30% SpCO. Patient 3 complained of a diffuse headache and was found to have a RAD 57 measurement of 21% SpCO.

## Methods

Howard County, Maryland is located between Baltimore and Washington, D.C. The county is 252 square miles in size and has a population of approximately 300,000. Emergency Medical Services are provided though Howard County Fire and Rescue (HCFR). HCFR is a combined career and volunteer department that responds to over 30,000 emergency calls per year. HCFR is the lead agency for Emergency Management in Howard County.

HCFR has encountered multiple previous cases of Carbon Monoxide toxicity in both departmental personnel as well as citizens during power outages and disasters.^10^ These cases included at least one fatality. Based upon these prior experiences, HCFR has implemented a public education intervention for the prevention of Carbon Monoxide poisoning into its Incident Action Plan (IAP) for events where electrical service interruptions are anticipated or have occurred.

In response to forecast data from the National Weather Service (NWS) leading up to Hurricane Sandy, Howard County Emergency Management (OEM) created an IAP. A Unified Incident Command System (ICS) was established which identified HCFR (including OEM) as lead agency for this incident. Other participating agencies included: Howard County Government Administration, Howard County Police Department, Howard County Department of Public Works and other agencies. Lines of communication between these agencies were coordinated through OEM, who communicated with the power company. These efforts were in addition to those enacted by State of Maryland through the State’s Maryland Emergency Management Agency, (MEMA).

Two topics were identified as focus areas for community education following Hurricane Sandy. These topics included education and prevention of carbon monoxide poisoning from portable generators, as well as risks associated with flooding. Public service announcement (PSA) documents were created and mass produced for distribution. Based upon the languages spoken in the community, this material was translated into three other languages (Spanish, Korean and Vietnamese)(Appendix 1).

A targeted prevention program was enacted that focused efforts on neighborhoods determined by the electrical utility provider service, Baltimore Gas and Electric (BGE), to be without power. This intervention combined face-to-face education with the distribution of written materials from Centers for Disease Control and Prevention (CDC) on the hazards of CO poisoning. During the initial 24 hour period following the disaster, HCFR personnel went door-to-door in neighborhoods without power to assess for evidence of lights on and listened for the sound of generators running. These activities partially occurred in the evenings to assess for homes with lights on, indicating the possible use of a generator. Efforts were made to establish direct contact with residents. Residents were educated about the hazards of generator use and informational flyers were distributed.

## Results

At the peak of the power outage following Hurricane Sandy BGE reported 38,431 of its 119,000 (32%) customers in Howard County without power. 24 hours following the storm, 14,000 (12%) outages remained in Howard County. BGE supplied HCFR with geocoded data on outage locations. During that period following the disaster, HCFR personnel evaluated affected areas in a door-to-door campaign and distributed approximately 500 flyers (Appendix 2). When residents were not home, or direct contact could not be made, flyers were left in mailboxes / front doors.

External costs for the project included those associated with the mass production of the prevention materials. These costs were absorbed through HCFR's existing logistics / support services budget and were approximately $300.00. On-duty personnel were used to facilitate the distribution of flyers in between emergency calls, which allowed for overtime costs to be avoided.

No CO related fatalities were reported in Howard County following Hurricane Sandy. Following Hurricane Irene (the most recent storm prior to Hurricane Sandy), there was one CO related fatality.

## Discussion

Anytime a storm causes widespread loss of electrical power, the potential exists for hazards associated with portable generator use. These generators are designed for outdoors use only as they can create deadly carbon monoxide gas. Multiple reports exist in the literature describing carbon monoxide exposure following storms. 3 ^,^ 4 ^,^ 5 ^,^ 6

A successful just-in-time prevention program requires surveillance methods that identify and effectively target at risk populations. In the case of a CO poisoning prevention and generator safety education effort, it is essential to identify homes without power where portable generators might be in use. During both Hurricane Irene and the Derecho, minimal real time information was made available from the electrical utility regarding the locations of power outages. Prior to Hurricane Sandy, surveillance methods relied primarily on flyovers using the Police Department helicopter. This proved to be an effective means to identify neighborhoods without power. In the aftermath of the Derecho, key policy changes made at the state level required the utility company to supply counties with detailed outage information. This proved invaluable following Hurricane Sandy when information was made available to counties about addresses and neighborhoods without power. In addition, this negated the need for helicopter flyovers, which are especially dangerous in a power failure situation where typically illuminated hazards (towers, smokestacks, buildings, etc) may not be as apparent.

One major challenge in community outreach and education during power outages is the inability to reach at-risk individuals who have become accustomed to reliance on internet and smart phone technology. For homes without power, internet access was unavailable and smart phones were not able to be charged. Thus the typical methods of rapid, instantaneous access to information were not available. Emergency Managers found themselves having to devise a system to disseminate critical just-in-time information to citizens without access to battery powered radios.

## Conclusion

This paper describes the early and novel public health efforts by a Fire Department to engage in door-to-door community outreach proactively to prevent illness and death from unsafe generator use in the period immediately following a natural disaster. This represents one of the first reported examples of a community-based endeavor by a fire department to provide proactive interventions designed to prevent carbon monoxide illness. This process has also allowed for identification of a secondary emerging problem of how to effectively disseminate essential information to members of the community who have become who have become dependent upon electrical and smart phone technology to receive the majority of their information. These technologies may not be available if such devices cannot be charged during a power outage. New and different methods of community outreach are needed to effectively communicate with this at-risk population.
